# Myeloid differentiation factor-2/*LY96*, a potential predictive biomarker of metastasis and poor outcomes in prostate cancer: clinical implications as a potential therapeutic target

**DOI:** 10.1038/s41388-023-02925-x

**Published:** 2023-12-23

**Authors:** Marina G. Ferrari, Alexis P. Jimenez-Uribe, Li Wang, Luke H. Hoeppner, Paari Murugan, Eunsil Hahm, Jindan Yu, Timothy M. Kuzel, Sergio A. Gradilone, Adrian P. Mansini

**Affiliations:** 1 Department of Urology, Rush University Medical Center, Chicago, IL USA; 2 Department of Internal Medicine, Rush University Medical Center, Chicago, IL USA; 3 The Hormel Institute, Masonic Cancer Center, University of Minnesota, Austin, MN USA; 4 Department of Laboratory Medicine and Pathology, University of Minnesota, Minneapolis, MN USA; 5 Department of Urology and Department of Human Genetics, Emory University School of Medicine, Atlanta, GA USA; 6 Winship Cancer Institute of Emory University, Atlanta, GA USA; 7 Department of Internal Medicine, Division of Hematology, Oncology and Cell Therapy, Rush Medical College, Chicago, IL USA; 8 The Hormel Institute, University of Minnesota, Austin, MN USA

**Keywords:** Prognostic markers, Tumour biomarkers

## Abstract

Prostate cancer (CaP) is the most diagnosed cancer in males and the second leading cause of cancer deaths. Patients with localized tumors are generally curable. However, no curative treatment exists for patients with advanced and metastatic disease. Therefore, identifying critical proteins involved in the metastatic process would help to develop new therapeutic options for patients with advanced and aggressive CaP. We provide strong evidence that Myeloid differentiation factor-2 (MD2) plays a critical role in metastasis and CaP progression. Analysis of tumor genomic data showed that amplifications of MD2 and increased expression are associated with poor outcomes in patients. Immunohistochemistry analysis of tumor tissues showed a correlation between the expression of MD2 and cancer progression. The Decipher-genomic test validated the potential of *MD2* in predicting metastasis. In vitro studies demonstrated that MD2 confers invasiveness by activating MAPK and NF-kB signaling pathways and inducing epithelial–mesenchymal transition. Furthermore, we show that metastatic cells release MD2 (sMD2). We measured serum-sMD2 in patients and found that the level is correlated to disease extent. We determined the significance of MD2 in metastasis in vivo and as a therapeutic target, showing that the molecular and pharmacological targeting of MD2 significantly inhibited metastasis in murine models. We conclude that MD2 predicts metastatic behavior, and serum-MD2 could be studied as a potential non-invasive biomarker for metastasis, whereas MD2 presence on prostate biopsy predicts adverse disease outcome. We suggest MD2-targeted therapies could be developed as potential treatments for aggressive metastatic disease.

## Introduction

Prostate cancer (CaP) is the most common cancer diagnosed in males, and is the second most common cause of cancer-related deaths [1]. Although patients with localized CaP are generally curable, exhibiting a 10-year overall survival rate of over 99% [2], patients with metastatic disease are incurable, metastasis being the major cause of mortality [3–9]. Currently, there is no curative treatment for patients with metastasis. Therefore, understanding mechanisms that confer metastatic potential to localized tumors is essential for developing novel biomarkers of cancer progression and therapies to inhibit the disease progression.

Metastasis involves multiple steps, including neovascularization and acquiring an invasive phenotype characterized by the expression of the epithelial–mesenchymal transition (EMT) markers [10]. Expression of the EMT markers represents a crucial step in the CaP progression, and targeting EMT would likely improve the overall survival of patients. Therefore, unveiling the molecular mechanisms responsible for EMT will allow us to develop new therapeutic options for metastatic CaP.

Myeloid differentiation factor-2 (MD2) (*LY96* gene) is a small glycoprotein expressed by macrophages and dendritic cells [11]. MD2 functions as a co-receptor for toll-like receptor (TLR) 4 and is required for its activation [12]. The TLR4 signaling pathway is involved in the oncogenesis of several cancers including CaP [13]. The expression of TLR4 and its activation are associated with CaP progression [14, 15]. Since MD2 is essential in TLR4 signaling, targeting MD2 may be a potential therapeutic approach for treating patients. However, no studies have shown the expression or importance of MD2 in CaP.

In this study, we provide evidence that CaP cells produce and release MD2 during cancer progression, which results in constitutive activation of the MAPK and NF-κB signaling pathways. We also show that MD2 is an essential factor in the tumor microenvironment allowing CaP cells to acquire metastatic traits. Therefore, we speculate that *MD2*-addicted tumor cells are prone to metastasis. Aided by the Decipher-genomic test, we also provide evidence about the potential use of MD2 as a predictive biomarker of patient disease outcomes. In addition, we determined the significance of MD2 in metastasis and as a therapeutic approach using murine models of lung metastasis.

## Results

### Alterations in the *MD2* gene in prostate cancer correlate with poor survival in patients

**Fig. 1:**
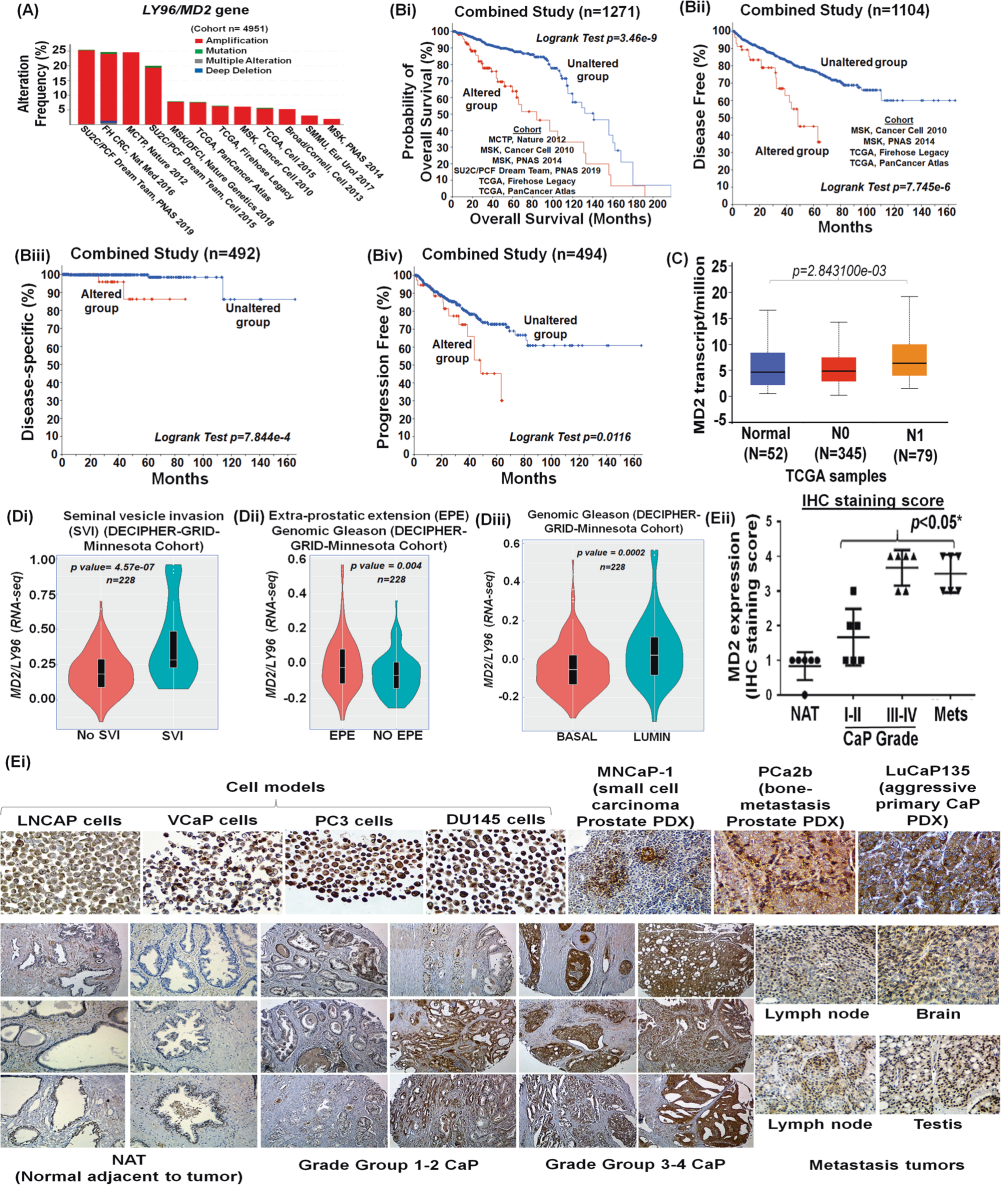
Relevance of MD2/LY96 alteration as a predictive biomarker of prostate cancer progression and poor survival in human patients. **A** The histogram shows the alteration frequency of the *MD2/LY96* gene in 12 clinical studies comprised of 4,951 patients. The data shows the different *MD2* gene alterations in the patients’ tumors. The data was analyzed from TCGA data sets. **Bi** Kaplan–Meier graph shows the analysis of TCGA clinical data establishing a correlation between MD2 alterations and overall survival, **Bii** Disease progression-free survival, **Biii** disease-specific survival, and **Biv** progression-free survival. The data were generated from tumor genome analysis of patients using the cBioportal platform. **C** The graph shows the expression of MD2 transcript in TCGA-PRAD samples classified as normal, N0: no regional lymph-node metastasis, and N1: metastases in 1 to 3 axillary lymph nodes. *Decipher-genomic test*. The graph shows the potential of the biopsy-MD2 alteration as a biomarker predicting the risk of seminal vesicle invasion (**Di)**, extraprostatic extension **(Dii)**, and genomic Gleason (**Diii)** by prostate tumor cells in CaP patients. *Immunohistochemistry*. **Ei** The expression of MD2 in prostate cancer cell-based models, patient-derived xenografts (PDX) models, one bone-metastasis cell-derived tumor xenograft and normal regions adjacent to tumor (NAT), primary prostate tumors with different grade groups, and metastatic tumors of CaP patients. **Eii** Staining score of the tissues assessed by IHC.

Since there is a strong association between cancer progression and chronic inflammation [16–18], we asked if MD2 is involved in CaP; therefore, we studied the association of the presence of *MD2* to the survival of patients in a large patient cohort, with survival and follow-up details available. We performed a comprehensive analysis of the tumor genome data of patients using the cBioPortal web platform. First, the genomic analysis of tumors of 4,951 CaP patients from 12 clinical studies suggested that the *MD2* gene exhibits a high frequency of alterations at the genomic level, particularly amplification of the gene (Fig. 1A and Table 1). We also observed a small number of patients who exhibited mutations or multiple alterations in the gene. In addition, to the genomic alterations, we identified that the most common alteration at the expression level was the high expression of the gene. Only four patients from two studies showed low expression of MD2 **(**Table 1**)**. Then, we analyzed the overall survival of patients with *MD2* alterations. We analyzed a cohort of 1,271 patients from 6 clinical studies, including 115 cases with alterations in *MD2* and 1,156 with no alterations. We found that cases with alterations exhibit a significantly lower overall survival (Log-rank Test *p* = 3.46e−9) than cases without alterations (Fig. 1Bi). The median overall survival for *MD2*-altered patients was 84 months, whereas subjects without alterations exhibited an overall survival of 141 months (Fig. 1Bi). Next, we analyzed the disease-free, disease-specific, and progression-free survival in combined studies. The results showed that patients with alterations exhibit significantly lower disease-free, disease-specific, and disease progression-free survival than patients without alterations (Log-rank Test *p* = 7.745e−6, *p* = 7.844e−4, *p* = 0.0116) (Fig. 1Bii–iv). Finally, we used the ULCAN platform and the PRAD-TCGA data to study a potential association between the *MD2* transcript levels in prostate tumors and metastasis in patients. Clinical data from 424 patients with various stages of prostate tumors including N0: no regional lymph-node metastasis (*n* = 345), and N1: metastases in 1 to 3 axillary lymph nodes (*n* = 79) was compared with 52 controls showed significantly higher levels of the *MD2* transcript in tumors with lymph-node metastasis (Fig. 1C). Thus, survival data sets indicate that *MD2* amplification and increased expression are associated with poor survival in patients.

**Table 1:** Alterations in the LY96/MD2 gene in clinical studies.

				Alteration detail					
				mRNA expression (%)		Genomic (%)			
Study	Samples	Patients	Alteration frequency LY96 (%)	Low	High	Deletion	Amplification	Mutation	MALT
Metastatic Prostate Adenocarcinoma (MCTP, Nature 2012)	61	59	24.59	–	–	–	24.59 (15)	–	–
Metastatic Prostate Adenocarcinoma (SU2C/PCF Dream Team, PNAS 2019)	444	429	25.45	–	–	–	25 (111)	0.23 (1)	0.23 (1)
Metastatic Prostate Cancer (SU2C/PCF Dream Team, Cell 2015)	150	150	20	–	–	–	19.33 (29)	0.67 (1)	–
Prostate Adenocarcinoma (Broad/Cornell, Cell 2013)	56	56	5.36	–	–	–	5.36 (3)	–	–
Prostate Adenocarcinoma (Broad/Cornell, Nat Genet 2012)	20	20	5	–	5 (1)	–	–	–	–
Prostate Adenocarcinoma (Fred Hutchinson CRC, Nat Med 2016)	133	54	24.81	0.75 (1)	1.5 (2)	–	20.3 (27)	0.75 (1)	1.5 (2)
Prostate Adenocarcinoma (MSK, Cancer Cell 2010)	126	126	15.08	2.38 (3)	6.35 (8)	–	5.56 (7)	–	0.79 (1)
Prostate Adenocarcinoma (MSK/DFCI, Nature Genetics 2018)	1013	1013	7.9	–	–	–	7.7 (78)	0.2 (2)	–
Prostate Adenocarcinoma (SMMU, Eur Urol 2017)	65	65	6.15	–	3.08 (2)	–	3.08 (2)	–	–
Prostate Adenocarcinoma (TCGA, Cell 2015)	333	333	9.91	–	4.2 (14)	–	4.8 (16)	0.3 (1)	0.6 (2)
Prostate Adenocarcinoma (TCGA, Firehose Legacy)	501	500	12.22	–	5.81 (26)	–	5.21 (26)	0.2 (1)	1 (5)
Prostate Adenocarcinoma (TCGA, PanCancer Atlas)	488	488	13.52	–	5.74 (28)	–	5.94 (29)	0.2 (1)	1.64 (8)
Prostate Adenocarcinoma Organoids (MSK, Science 2022)	12	7	8.33	–	8.33 (1)	–	–	–	–

*MALT* multiple alterations.

### *MD2* as a potential predictive biomarker

Decipher test provides multiple algorithms to predict the clinical outcomes of CaP patients based on genome data of primary tumors. Based on a 22-gene signature, the basic Decipher test classifies patients as low, average, and high for therapy outcome or risk of recurrence or metastasis [19]. The predictive accuracy of the test can be made more robust by adding new algorithms such as the Genomic Gleason and CAPRAS algorithms; we asked if MD2 as a marker could identify locally invasive tumors prone to recur. We previously reported utilizing the Decipher test in a cohort of 228 patients that biopsy-S100A4 overexpression predicts poor ADT response and a high risk of mortality [20]. We used the same data cohort to study the association of MD2 with CaP progression, where the patients were classified as low, average, or high by the test. Seminal vesicle invasion (SVI) and extraprostatic extension (EPE) are validated indicators of poor outcomes and adverse prognosis in patients [21–23]. SVI is associated with increased likelihood of local recurrence and development of future metastasis and upstages CaP to stage III (pT3a and pT3b) CaP [24]. Our exploratory cohort was classified as “No SVI” and “SVI” by the test. Our data showed that *MD2*-high expression is significantly correlated to SVI-positive cases. We found that *MD2*-high expression cases are significantly (*P* = 4.57e−07) identifiable with Decipher-classified SVI cases (Fig. 1Di).

Extraprostatic extension (EPE) describes a tumor stage where the tumor extends beyond the prostate borders and is also associated with increased risk of metastasis [22, 25]. Patients in whom EPE is detected on prostate biopsy are considered to have adverse pathologic finding after RP [22]. The Decipher-algorithm predicts the risk of EPE. The test identified EPE cases in the cohort and classified them as EPE positive and NO EPE. When these cases were tested for *MD2* expression, a significant correlation (*p* = 0.0004) between high-*MD2* expression with EPE positivity was observed (Fig. 1Dii). These data support the notion that increased expression of *MD2* is associated with pathologic features of localized tumors which have increased risk for local and distant recurrence.

Decipher has developed multiple other cancer-subtype algorithms, which allow for finding common markers between sub-types and metastasis. The two outputs of the algorithm are “basal” and “lumen,” thus determining if a protein’s sub-tissue distribution affects the metastasis. Most metastatic prostate tumors are from the luminal region in prostate. The Decipher test classified the patient cohort as basal and luminal. The analysis showed that high-*MD2* cases are significantly (*p* = 0.0002) predicted to be of luminal subtype CaP, whereas low-*MD2* cases could be basal type (Fig. 1Diii). Thus, multiple algorithms suggested that the expression of *MD2* is associated with poor clinical outcomes.

These findings in a Decipher cohort establish the correlation of MD2 with advanced disease and metastasis.

### MD2 protein levels in prostate tumors

Aggressive tumor cells in the tumor microenvironment (TME), while getting addicted to certain factors present in the TME, also start expressing such factors themselves. MD2 is mainly found in immune cells. However, its presence in prostate tumors has not yet been reported. Therefore, we performed the IHC analysis to determine the presence of MD2 in CaP tissues. First, we performed antibody validation and specificity testing (Supplementary data) by IHC analysis of Histogel-embedded metastatic CaP (mCaP) cell models (LNCaP, VCaP, PC3, and DU145). We found that the antibody detected MD2 in CaP cells and differentiated between MD2-rich cells from the MD2-deficient cell model. This is evident from the data where MD2 was found to be highly expressed in VCaP, PC3, and DU145 whereas it is scantily present in LNCaP (Fig. 1Ei). It is to be noted that VCaP, PC3 (bone metastasis-derived), and DU145 (brain metastatic-tumor derived) are considered highly aggressive cells, whereas LNCaP is a slow-growing lymph-node-derived cell line. Second, we evaluated the expression of MD2 in two primary patient-derived xenografts (PDX) models and one bone-metastasis cell-derived tumor xenograft. These included LuCaP135 (primary invasive tumor), MNCaP1 (aggressive primary prostate small cell carcinoma), and PCa2b (PCa2b cell-derived bone tumor). The IHC analysis confirmed the presence of MD2 in all PDX tumors (Fig. 1Ei).

Next, we evaluated the expression of MD2 in NAT (normal tissues adjacent to the cancerous region), primary (Grade Groups (GG) I–II or GG III-IV), and metastatic tumors (lymph-node, brain, testis). The IHC analysis of patient specimens showed that prostatic tumors exhibit elevated immunostaining compared to the NAT (Fig. 1Ei) (Supplementary data). Notably, metastatic tumors exhibited more positive immunostaining for MD2 than primary tumors (Fig. 1Ei, Eii). The immunostaining intensity was scored on a scale of 0–4 (0 = none, 1 = weak/scant, 2 = moderate, 3 = strong, 4 = highly strong). Based on the immunostaining score, we compared the expression of MD2 between tumor grades. In comparison, MD2 expression in primary tumors of GG I–II (*p* < 0.05) and GG III–IV *(p* < 0.001) was significantly higher than NAT (Fig. 1Eii). The expression in GG-III/IV was almost equal to metastatic tumors (Fig. 1Eii). Out of all metastatic tumors, metastatic brain tumors exhibited higher MD2-immunopositive cells (Fig. 1Eii). These data show that MD2 is highly expressed in GGIII/IV primary and metastatic tumors and that MD2 levels increase progressively in patients during disease progression.

We asked if the increment of the MD2 during CaP progression is a translational event or originates at the transcriptional level. For this, we performed qPCR analysis of human primary prostate tumors, metastatic tumors, and NAT. Metastatic tumors exhibited a higher level of *MD2* transcript than primary tumors (*p* < 0.05). Notably, some metastatic tumors showed *MD2* like primary tumors (Supplementary Fig. Ai). These data suggest that an increase in MD2 occurs during progressive phases of CaP and suggest MD2 as an indicator of disease progression in patients.

### MD2 expression in a cell-based progression model

**Fig. 2:**
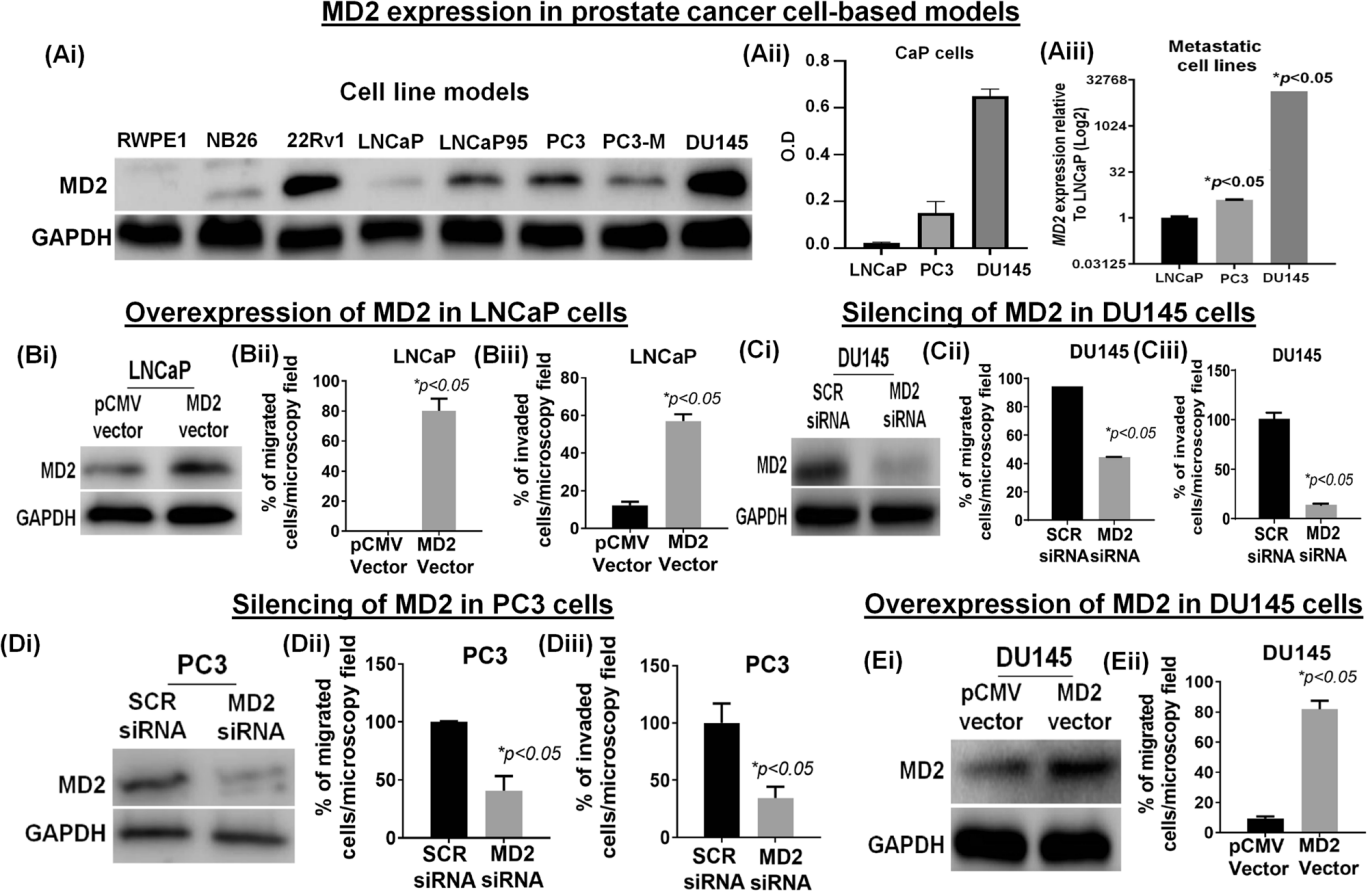
Relevance of MD2 for metastasis of the prostate cancer cell lines. **Ai** Immunoblot image shows the protein level of MD2 in CaP cell models representing normal (RWPE1), premalignant/indolent PCa (RW-NB26), primary PCa (22Rv1), lymph-node metastasis (LNCaP), AR-positive CRPC (LNCaP95), bone-metastasis (PC3 and PC3-M), and brain-metastasis (DU145), assessed by immunoblot analysis. The GAPDH protein levels in cell lysates were used as a loading control. **Aii** Quantification of MD2 by densitometry in LNCaP, PC3, and DU145 cells. **Aiii** Expression of mRNA MD2 in LNCaP, PC3, and DU145 cells assessed by RT-qPCR. **Bi** Immunoblot image shows the expression of MD2 in LNCaP expressing the MD2 vector. **Bii-iii** Histograms compare the migratory (**Bii**) and invasive (**Biii**) potential of LNCaP cells expressing the MD2 vector. **Ci**, **Di** Immunoblot images show the MD2 suppression in PC3 and DU145 cells. **Cii–iii**, **Dii–iii** Histograms compare the migratory (**Cii**, **Dii**) and invasive (**Ciii**, **Diii**) potential of PC3 and DU145 cells silencing MD2. **Ei** Immunoblot image shows the overexpression of MD2 in DU145 cells and **Eii** its effect on migration.

Although we showed the expression of MD2 in a few CaP cell models (Fig. 1Ei), we expanded our examination to a spectrum of CaP cell lines. We evaluated the level of MD2 in immortalized normal prostate epithelial cells (RWPE1), primary indolent (NB26), primary-CRPC (22RV1), androgen-dependent metastatic (LNCaP), and mCRPC (LNCaP95, PC3, PC3-M, and DU145) models by immunoblotting. The results showed that MD2 is significantly high in CRPC cells, particularly in metastatic cells (Fig. 2Ai). The exception was LNCaP which exhibited scant expression of MD2, which corroborated with our IHC data (Fig. 1Ei). The analysis by densitometry shows an expression level in DU145 > PC3 > LNCaP (Fig. 2Aii). Next, we compared the *MD2* transcript in DU145, PC3, and LNCaP cells and found that cells with high-MD2 protein harbor a high level of *MD2* transcript (*p* < 0.05) (Fig. 2Aiii). Additionally, we evaluated the expression of the MD2 protein in the neuroendocrine CaP cell line NCI-H660 and found weak expression (Supplementary Fig. Aii). These data show that MD2 is associated with advanced CaP in a progressive cell model and further cement the position of MD2 as a potential biomarker of CaP progression and metastasis.

### Intracellular expression of MD2 induces migration and invasion

Since LNCaP exhibit a lower expression of MD2 and a lower metastatic potential than PC3, and DU145, we evaluated the significance of the MD2 in these metastatic cells. Thus, to study the role of MD2 in CaP progression, we focused on LNCaP, PC3, and DU145. To investigate the role of MD2 in metastasis, we overexpressed MD2 in LNCaP. We confirmed the overexpression by immunoblotting (Fig. 2Bi) and immunofluorescence (Supplementary Fig. Aiii) and evaluated the viability, migration, and invasion. We found that the ectopic overexpression of MD2 did not alter the proliferation rate assessed by MTT and by immunoblotting, evidenced by the expression of PCNA (Supplementary Fig. Bi, ii). However, MD2 regulated the migration and invasion of these cells. The overexpression of MD2 results in an enhanced migration and invasion compared with controls (Fig. 2Bii, iii). Then, we silenced MD2 in PC3 and DU145 (MD2 siRNA) (Fig. 2Ci, Di) and evaluated the viability, migration, and invasion. As expected, the silencing did not modify the cell growth (Supplementary Fig. Biii, iv). However, the silencing inhibited migration and invasion (Fig. 2Cii, iii, Dii, iii). Furthermore, we observed that the silencing of MD2 in the 22RV1 cell line also decreased the invasive potential of the cells (Supplementary Fig. Ci–iii). Complementary, we overexpressed MD2 in DU145 cells (Fig. 2Ei), which exhibit a high basal level of MD2, and then assessed migration. The result showed that DU145 overexpressing MD2 display a higher rate of migration than the control (Fig. 2Eii) (Supplementary Fig D). Finally, we evaluate the effect of the modulation of MD2 in LNCaP and DU145 on the potential of the cells to cross the vascular barrier (layer of human endothelial cells (HUVEC)) using an in vitro model of extravasation. The overexpression of MD2 in LNCaP induced a significant increment in the number of transmigrated cells (Supplementary Fig. Ei), to the contrary, silencing of MD2 in DU145 caused a significant decrease (Supplementary Fig. Eii). These results suggest MD2 confers metastatic behavior characterized by increased migration and invasion.

### Intracellular expression of MD2 induces NF-κB signaling

**Fig. 3:**
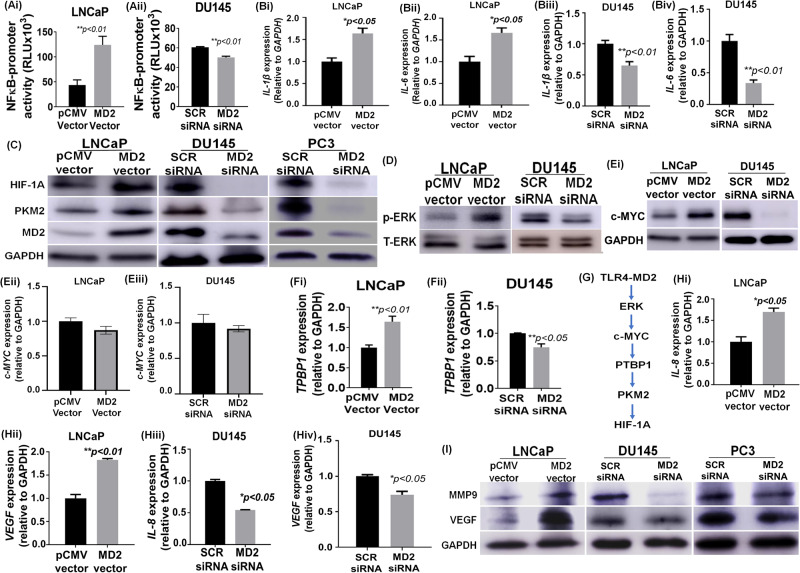
MD2 signaling pathways. **Ai–ii** Histograms compare the NF-κB promoter activity in LNCaP overexpressing MD2 and DU145 silencing MD2 assessed by dual-luciferase reporter assays. Each bar of histograms represents the average of three independent experiments. Renilla luciferase activity served as the internal control for each group. **Bi–iv** Histograms show the effect of the MD2 overexpression in LNCaP cells or MD2 silencing in DU145 cells on IL-1β and IL-6 mRNA assessed by RT-qPCR. **C** Immunoblot images show the effect of MD2 overexpression (LNCaP) or MD2 suppression (DU145 and PC3) on HIF-1A and PKM2 expression. **D** Immunoblot images show the effect of MD2 overexpression or suppression on phospho- and total-ERK proteins. **Ei–iii**, **Fi**–**ii** Effect of MD2 overexpression or suppression on c-MYC protein assessed by immunoblotting and c-MYC and TPBP1 mRNA assessed by RT-qPCR. **G** MD2 downstream targets model suggested. **Hi–iv** Histograms show the effect of the MD2 overexpression in LNCaP cells or silencing in DU145 cells on IL-8 and VEGF mRNA assessed by RT-qPCR. **I** Immunoblot images show the effect of MD2 overexpression (LNCaP) or suppression (DU145 and PC3) on the precursor form of MMP9 and VEGF proteins.

To explore the mechanisms underlying the MD2-dependent metastatic observation we focused on the TLR4 signaling pathway because MD2 is required for its activation. Thus, we measured the NF-κB promoter activity in LNCaP overexpressing MD2 and DU145 silencing MD2. We found that the overexpression of MD2 induced strong activation of the NF-κB promoter activity (Fig. 3Ai), and to the contrary, silencing of MD2 in DU145 resulted in a lower level of activity (Fig. 3Aii). In addition, we assessed the expression of p65, which is increased in cells with sustained activation of NF-κB signaling. We found that in cells overexpressing MD2, p65 was higher than the control. Contrarily, the cells silencing MD2 exhibit a lower expression (Supplementary Fig. F).

Then, we evaluated the expression of the downstream targets of TLR4, namely IL-1β, and IL-6 by qPCR. We found that LNCaP overexpressing MD2 exhibit high levels of both interleukins (Fig. 3Bi, ii). On the contrary, the silencing of MD2 in DU145 resulted in a lower expression of IL-1β and IL-6 (Fig. 3Biii, iv). These data show that the expression of MD2 in metastatic CaP cells induces pro-inflammatory cytokines through the activation of NF-κB signaling.

### Intracellular expression of MD2 regulates HIF-1A and PKM2

The activation of TLR4 by some ligands results in the induction of HIF-1A and PKM2 via the activation of NF-kB in macrophages [26]. However, the consequences of its activation in CaP cells are unknown. Therefore, we evaluated if the expression of MD2 induces HIF-1A and PKM2 in CaP cells. For this, we assessed the levels of HIF-1A and PKM2 by qPCR and immunoblotting in cells overexpressing and silencing MD2. The results showed that overexpression or silencing of MD2 did not modify the levels of these mRNAs (Supplementary Fig. Gi–iv); however, we found a significant change in the protein levels. LNCaP overexpressing MD2 exhibit a strong expression of HIF-1A and PKM2 (Fig. 3C), while silencing of MD2 in PC3 and DU145 resulted in a marked decrease of the expression of both proteins (Fig. 3C). It is well documented that HIF-1A and PKM2 exhibit positive feedback, and PKM2 can bind HIF-1A protein inducing its stabilization [27]. Therefore, we investigated upstream PKM2 proteins. Since activation of TLR4 results in ERK phosphorylation that induces stabilization of c-MYC, we evaluated phospho-ERK in cells overexpressing and silencing MD2 and its possible role in c-MYC stabilization. We found that LNCaP overexpressing MD2 exhibit strong phosphorylation of ERK (Fig. 3D). On the contrary, silencing MD2 in DU145 decreased the phosphorylation (Fig. 3D). Furthermore, we found that the high level of phospho-ERK was associated with high levels of c-MYC protein. However, we did not find any change in the mRNA expression levels (Fig. 3Ei–iii). c-MYC induces the expression of PTBP1, which causes the switch from PKM1 to PKM2 [28]. Therefore, we assessed the levels of *PTBP1* by qPCR. The result showed that cells overexpressing MD2 exhibit a significant increment of *PTBP1* (Fig. 3Fi), while cells silencing MD2 exhibit lower levels (Fig. 3Fii). These data suggest that MD2 induces activation of ERK and stabilizes c-MYC at the protein level, which in turn may induce an increment in the level of PKM2 protein via induction of PTBP1, leading to HIF-1A stabilization (Fig. 3G).

### Intracellular expression of MD2 induces IL-8 and VEGF

IL-8 expressed by CaP cells promotes metastasis and aggressiveness [29]. Since it was reported that HIF-1A mediates the induction of IL-8 and VEGF [30], we evaluated if the overexpression of MD2 results in higher levels of IL-8 and VEGF. Therefore, we assessed the levels of *IL-8* and *VEGF* transcripts in cells overexpressing and silencing MD2. The results showed that LNCaP overexpressing MD2 exhibited a high level of *IL-8* and *VEGF*, while silencing of MD2 in DU145 resulted in a decrease in the level of these transcripts (Fig 3Hi–iv). These data suggest that MD2 may regulate the expression of *IL-8* and *VEGF* in metastatic CaP cells, possibly through the induction of HIF1-A.

### Intracellular expression of MD2 induces VEGF and MMP9

Due to mCaP cells expressing high levels of MMP9 required for metastasis and VEGF participating in a positive feedback regulation between MMP9 and VEGF, we evaluated the effect of MD2 on the expression of the precursor form of MMP9 and VEGF by immunoblotting. The results showed that the overexpression of MD2 in LNCaP induced a significant increment of MMP9 and VEGF. To the contrary, in PC3 and DU145 cells, MD2 silencing resulted in levels of MMP9 and VEGF that were lower than controls (Fig. 3J). All these data show that MD2 regulates several signaling pathways involved in metastasis and confers aggressive characteristics to CaP cells.

### Silencing of MD2 attenuates epithelial–mesenchymal transition

**Fig. 4:**
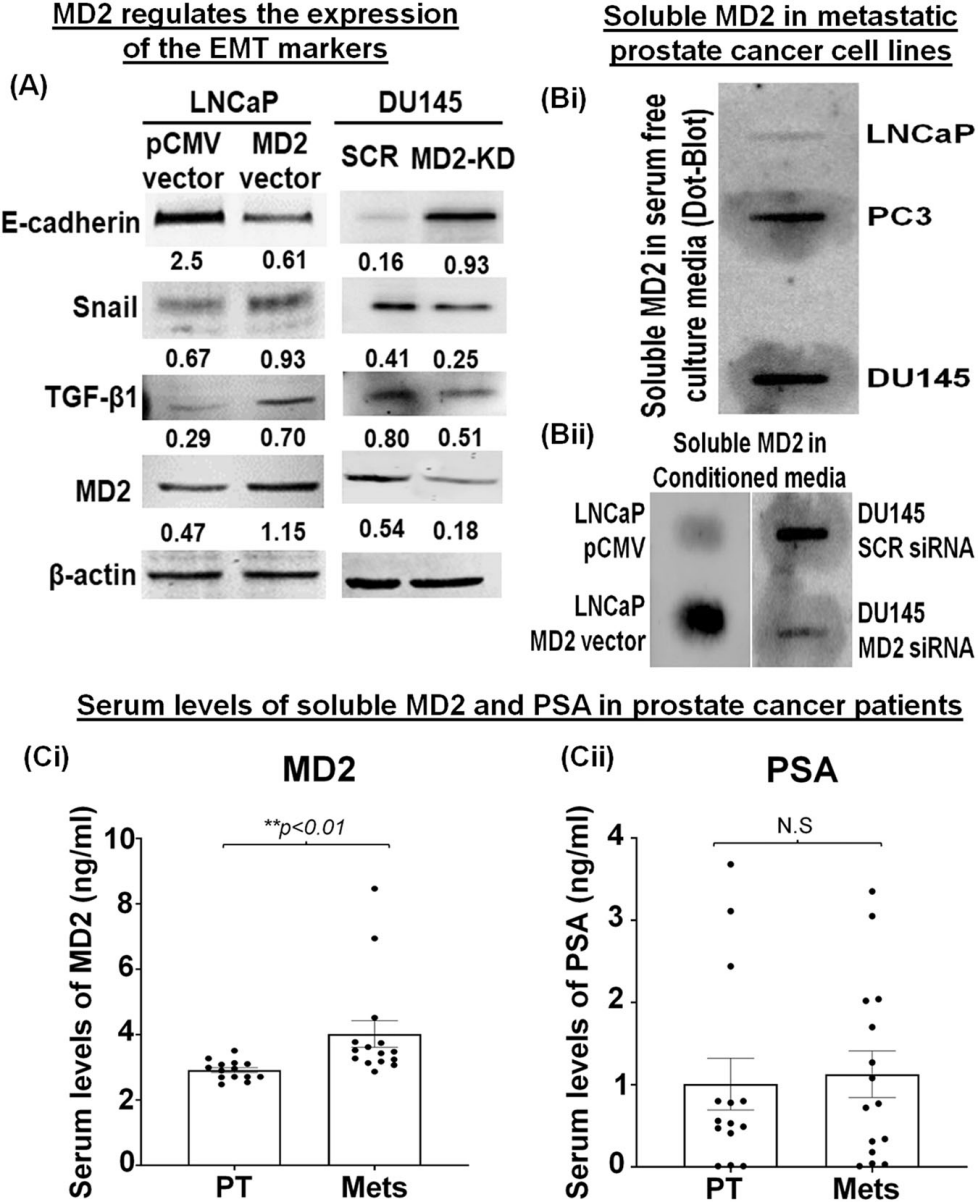
MD2 induces the expression of the EMT markers and the potential use of soluble MD2 as a biomarker of metastasis. **A** Immunoblot images show the effect of overexpressing MD2 in LNCaP and silencing MD2 in DU145 cells on the expression of EMT markers. **Bi–ii** Dot-blot images show the absence or presence of soluble MD2 in conditioned media of LNCaP, PC3, and DU145 cells and the effect of MD2 overexpression or suppression on soluble MD2. **Ci–ii** Serum levels of soluble MD2 and PSA in prostate cancer patients diagnosed with primary tumors (PT) or metastasis (Mets) assessed by ELISA.

Because we found that MD2 regulates critical proteins involved in the metastatic process and considering that EMT is a major hallmark of metastatic disease, we evaluated the expression of the mature secreted form of the TGF-β1 protein, the principal EMT inducer in PCa [31, 32]. We found that overexpression of MD2 in LNCaP resulted in increased expression of TGF-β1 compared with the control (Fig. 4A). We also found that the epithelial marker E-cadherin was decreased. At the same time, the mesenchymal marker Snail was increased (Fig. 4A). Then, we evaluated the expression of the mature secreted form of the TGF-β1 protein, E-cadherin, and Snail in DU145 cells stably transfected with shRNA-MD2. The immunoblot showed that the downregulation of MD2 decreased the expression of TGF-β1 and Snail while increasing E-cadherin (Fig. 4A). Furthermore, we observed that the silencing of MD2 in DU145 cells reduced the levels of the mesenchymal markers N-cadherin and vimentin (Supplementary Fig. H). These data suggest that MD2 is directly associated with and regulates the EMT process, and its neutralization could be studied as a novel therapeutic approach.

### Soluble MD2 as a potential biomarker for advanced CaP

To study MD2 as a potential biomarker, we asked if mCaP cells expressing MD2 release the soluble form (sMD2) into the microenvironment. We first assessed the localization of MD2 in LNCaP overexpressing MD2 by IHC. We found that in LNCaP, the expression was very poor, and the localization was focal in the interior of the cells. In contrast, in LNCaP overexpressing MD2, the expression was very intense. The distribution was focal in the interior of the cells, with robust staining in the membrane and outside the cells (Supplementary Fig. I), which suggests that MD2 may be released outside the cells. To evaluate this possibility, we assessed the presence of sMD2 in the conditioned media from LNCaP, PC3, and DU145 by dot-blot. We found that sMD2 was detected only in highly metastatic cells (PC3 and DU145) (Fig. 4Bi). Then, we evaluated the effect of overexpressing and silencing MD2 in LNCaP and DU145 on the release of sMD2. For this, we measured sMD2 in conditioned media from stables clones of LNCaP (pCMV and MD2 vector) and DU145 (DU145 SCR siRNA and MD2 siRNA). We found that sMD2 was detected in conditioned media from LNCaP overexpressing MD2 but not in the control (pCMV) (Fig. 4Bii). Conversely, the silencing of MD2 in DU145 resulted in a lower level of sMD2 in the conditioned media (Fig. 4Bii). Then, we evaluated sMD2 as a potential biomarker for advanced CaP. Therefore, we measured sMD2 in the serum samples of patients diagnosed with CaP by ELISA. We included 14 patients diagnosed with primary tumors (PT) and 16 with metastatic tumors (Mets). The results showed that the levels of sMD2 correlated with the progression of the disease. Metastatic patients exhibit a higher level of sMD2 with a mean of 4.0 ng/ml (2.7–8.4 ng/ml) compared with patients with PT with a mean of 2.9 ng/ml (2.4–3.5 ng/ml) (**p < 0.01) (Fig. 4Ci). Because we found that the levels of sMD2 are significantly different between patients with PT and Mets, we assessed the levels of PSA to evaluate if the levels of PSA also can differentiate PT of Mets in the same cohort of patients. The result showed that the levels of PSA could not differentiate PT from Mets. In patients with PT, the mean was 1.0 ng/ml (0.01–0.8 ng/ml), while in Mets was 1.1 ng/ml (0.01–3.3 ng/ml) (Fig. 4Cii). Thus, these data suggest that sMD2 may be further studied as a potential novel biomarker for advanced CaP disease. Thus, these data strongly suggest that sMD2 may be studied as a novel biomarker for advanced CaP disease.

### Cancer cell extravasation model of metastasis in zebrafish

**Fig. 5:**
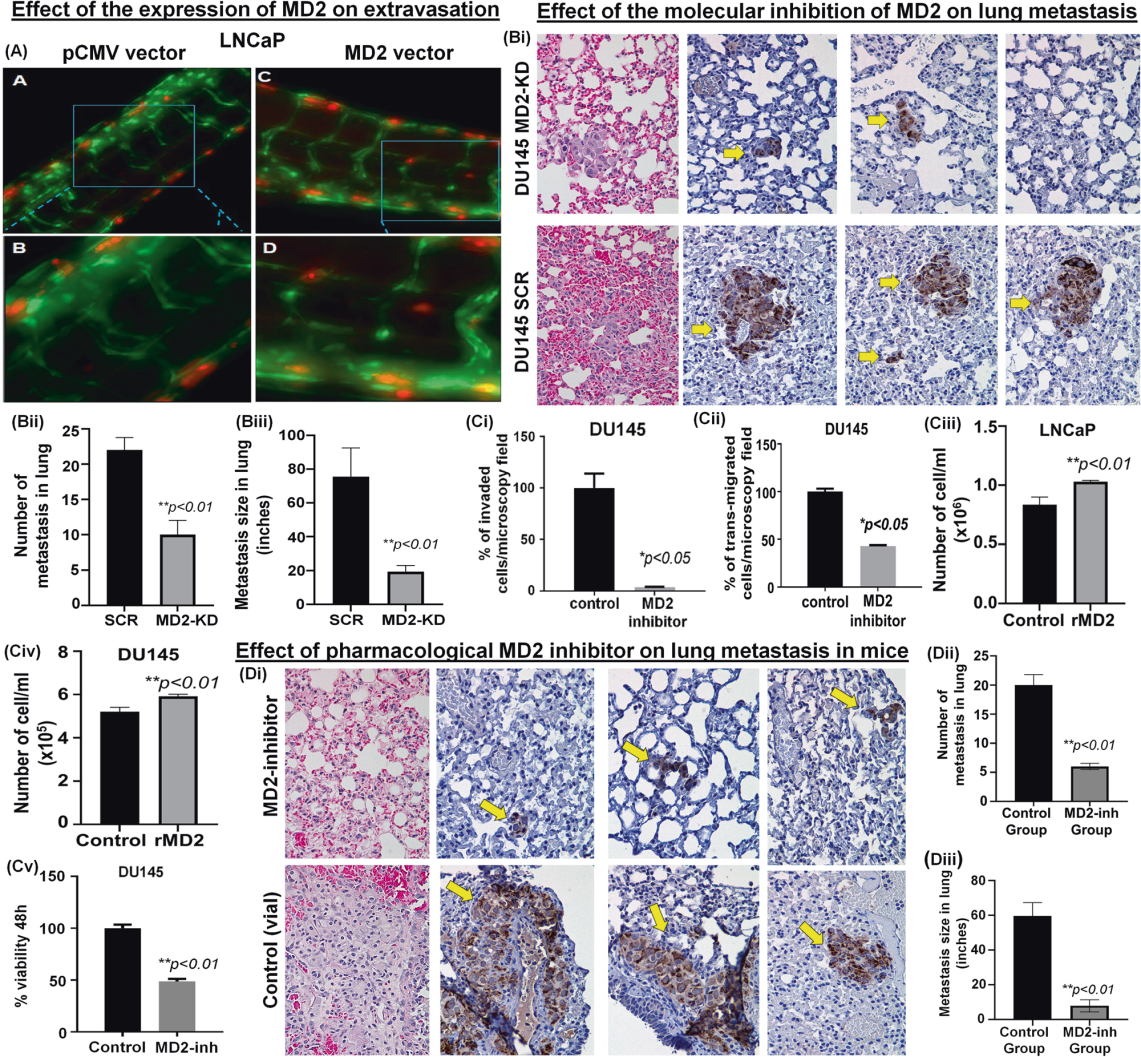
Targeting MD2 as a therapeutic approach. **A** Image shows the effect of overexpressing MD2 on the LNCaP cells extravasation in a Zebrafish in vivo model for metastasis. LNCaP cells were transiently labeled with a fluorescent tracker dye (red) and the bloodstream in green (GFP). **Bi** Effect of the molecular inhibition of MD2 on lung metastasis. Images compared the hematoxylin-eosin and anti-mitochondria lung tissue staining in control (SCR-shRNA) and MD2-Knockdown (MD2-KD) mice. Yellow arrows show tumor cells in the lung tissue of mice assessed by IHC staining using anti-human mitochondria antibody. **Bii**–**iii** Bar graphs show the number and size of metastasis in the control (SCR) and MD2-KD group of mice. **Ci**–**ii** Bar graphs show the effect of the pharmacological targeting MD2 using a small molecule inhibitor (10 µM) in DU145 cells on transmigration and invasion. **Ciii**–**vi** Bar graphs show the effect of recombinant human MD2 protein (rMD2) on proliferation assessed by cell counting. **Cv** The bar graph shows the effect of MD2 inhibitor (10 µM) on cell viability assessed in 48 h by MTT assay. **Di** Effect of pharmacological targeting MD2 in a mouse lung metastasis model. Images compared the hematoxylin-eosin and anti-mitochondria lung tissue staining in control and treated (MD2 inhibitor) mice. Yellow arrows show tumor cells in the lung tissue of mice assessed by IHC staining using anti-human mitochondria antibody. **Dii-iii** Bar graphs show the number and size of metastasis in the control and treated groups of mice.

During metastasis, tumor cells originating from the primary site migrate through the bloodstream and colonize distant locations. As part of this process, tumor cells invade and reside in non-primary sites by extravasating from the bloodstream. Zebrafish have emerged as an important vertebrate model to study cancer metastasis because they are amenable to in vivo imaging and share histological and genetic similarities with humans [33–36]. We have previously established a zebrafish xenotransplantation model of human cancer cell extravasation, which enables the visualization and assessment of extravasation events [37–39]. To assess the effect of MD2 in the extravasation and metastasis of human CaP cells, LNCaP stably overexpressing MD2 (and control) were transiently labeled with a fluorescent tracker dye and microinjected into the bloodstream 3 days post-fertilization Tg(fli:GFP) of zebrafish embryos via the pericardium. The following day at 24 h post injection of the cells, the larval zebrafish were imaged using a fluorescence microscope. We found that control LNCaP cells remained in the vasculature, whereas LNCaP stably overexpressing MD2 were in the extravascular space (Fig. 5A, Supplementary Fig. J). These in vivo results suggest that MD2 promotes the extravasation of human CaP cells, an important step required by metastasis.

### Inhibition of MD2 suppresses lung metastasis in a murine model

Since we found that MD2 provides metastatic properties to the cancer cells inducing EMT, and we found that the expression of MD2 in LNCaP resulted in increased extravasation, we evaluated the effect of the molecular silencing MD2 in DU145 on lung metastasis using a murine model. For this, we injected DU145 MD2 knocked-down cells stably transfected shRNA-MD2 or control (shRNA-SCR) via the tail vein in immunocompromised mice and evaluated the presence of the cancer cells in the lung 30 days later. Hematoxylin-eosin and IHC staining with anti-human mitochondria showed that silencing of MD2 significantly decreased the number and size of the metastasis per lung (Fig. 5Bi–iii).

Then, we studied the pharmacological inhibition of MD2 as a therapeutic approach. First, we evaluated the effect of the inhibition of MD2 in DU145 on the chemoinvasion and transmigration ability through HUVEC using MD2-int-1, a small molecule inhibitor of MD2. The results showed that the treatment significantly decreased both metastatic characteristics (Fig. 5Ci, ii). These results reinforce our hypothesis that MD2 is involved in metastasis in CaP for promoting and facilitating migration and invasion of the cancer cells.

Because we found that the inhibitor suppresses the transendothelial migration ability of the metastatic cells, we tested the inhibitory effect of the inhibitor in a mouse model of lung metastasis. First, we evaluate the effect of recombinant MD2 (rMD2) on cell growth in LNCaP and DU145. As shown (Fig. 5Ciii, Civ), rMD2 induced a moderate but significant increment in the number of cells assessed by counting. Then, we treated DU145 with the inhibitor and evaluated the viability by MTT. The results show that treatment resulted in a reduced percentage of viable cells compared with the control (Fig. 5Cv). Finally, we tested the efficacy of the therapy in lung metastasis in mice. We used DU145 cells to evaluate the lung metastasis after 30 days of tail vein injection. After treatment, the mice were euthanized, and the lugs were dissected and analyzed by Hematoxylin-eosin staining and IHC. As shown (Fig. 5Di), the hematoxylin-eosin staining shows that the treatment inhibited almost absolutely the presence of lung metastasis.

In comparison to the treated group, the control group exhibited substantial infiltration of tumor cells, which results in the loss of the normal shape and characteristics of the lung (Fig. 5Di). Furthermore, when we compared the number of metastases in both groups, we found that the group treated with MD2 inhibitor showed an average of 5 metastasis, while in the control group, the average of metastasis was 20 (Fig. 5Dii). In addition, we evaluated the presence of human mitochondria in the lungs by IHC. The result showed intense staining in lungs obtained from the control group, while a small focus was observed in the treated group (Fig. 5Di). Furthermore, the measure of the metastasis size in both groups was significantly different; while the average size in the control group was around 60 inches, in the treated group was 8 inches (Fig. 5Diii). All these data show that MD2 actively participates in lung metastasis in mice and is a druggable target to treat metastatic CaP.

## Discussion

The high rate of metastasis in CaP and the lack of curative therapy highlight the need to develop new and more efficient therapies [3–9]. Therefore, it is imperative to identify novels therapeutic targets to block the metastatic process and new biomarkers for cancer progression to personalize the treatment. It is recognized that patients with localized CaP often respond to primary therapy; however, a substantial number of men will develop metastasis [3–5]. Identifying novel biomarkers associated with CaP progression will help the clinician better guide therapy, perhaps choosing adjuvant therapies post-initial local therapy, and to better monitor/evaluate the progression of the treatment. This study assesses the significance of MD2 detection in patients and its role in CaP.

Since we found a strong association between the expression of MD2 and metastasis, we analyzed the significance of MD2 in the outcome of the patients by the analysis of a large patient cohort (TCGA-PRAD), where we found that alterations in *MD2* and increased expression are associated with the poor outcome. Although we observed a small number of patients exhibiting mutations or multiple alterations in the gene, most have amplification and high expression of *LY96/MD2*. Therefore, we understand that amplification and increased expression are associated with poor outcomes. Thus, the clinical data strengthens our hypothesis that MD2 is involved in aggressiveness, metastases, and CaP progression.

The current study delves into the role of MD2 as a potential predictive biomarker of metastasis and cancer progression in patients who undergo primary treatment after RP. The Decipher test is used to predict the outcome of the patients. Thus, by employing the test, we successfully validated the significance of the expression of *MD2* in patients who were predicted to develop metastasis and the worst outcome.

We provide evidence that metastatic cells express and release MD2 during cancer progression, providing the cell with increased migration and invasiveness potential. The metastatic characteristics were associated with the activation of the MAPK and NF-ΚB signaling pathways and by inducing EMT.

On the other hand, by using an in vivo extravasation model, we provide evidence that the expression of MD2 induces transendothelial tumor cell migration, which is essential for developing metastasis. Furthermore, in a lung metastasis model, we showed that the molecular silencing of MD2 significantly decreased the metastatic potential of the CaP cells. Additionally, we showed that MD2 is a druggable target for metastatic CaP. Therefore, MD2-targeted therapies, or therapies directed at its downstream protein products, could be developed as potential treatments for aggressive metastatic CaP.

Finally, in contrast to the PSA levels, which could not differentiate patients with PT from Mets, the levels of sMD2 correlated with the progression of the disease, thus highlighting the need to determine sMD2 in patients because it may represent a potential non-invasive biomarker for identifying advanced the disease. However, the small number of samples analyzed represents a limitation in this study. Therefore, further studies with more patient samples are needed to determine if determination of sMD2 can serve as a complementary biomarker of metastasis development and cancer progression.

In conclusion, MD2 expression on biopsy tissue would improve the performance of the Decipher-test in predicting the disease outcome in CaP patients. MD2 could represent a potential non-invasive new biomarker for advanced CaP, and detection in both biopsy specimens and serum may allow treating the patients with more aggressive early therapy to improve the outcomes and treat micrometastatic disease. Furthermore, therapies targeting MD2 may potentially treat aggressive CaP. For clinical use, MD2-targeting agents warrant a thorough investigation in animal models of CaP.

## Materials and methods

### Cell lines, antibodies, patient tissues, survival analysis, patient Cohort, transfections, and chemicals, Zebrafish in vivo model, lung metastasis models, and statistical analyses

Descriptions are provided in Supplementary methods.

### Cell growth, migration, chemoinvasion, and transmigration assays

These assays were performed as described previously [40–42].

### Confocal microscopy, immunoblot, IHC, RT-qPCR, luciferase assay

All the experiments were performed per published methods [40, 41, 43, 44].

### Supplementary information

Supplementary Figure

Supplementary methods

Supplemantary data

## Data Availability

All data generated or analyzed during this study are included in this published article and the supplementary files.
