# Supplementary material for: Myeloid differentiation factor-2/LY96, a potential predictive biomarker of metastasis and poor outcomes in prostate cancer: clinical implications as a potential therapeutic target
Source: Oncogene. 2023 Dec 23;43(7):484–94. doi: 10.1038/s41388-023-02925-x (PMC10857939; doi:10.1038/s41388-023-02925-x)
Supplement: Supplementary file 1 — Supplementary Figure [file 41388_2023_2925_MOESM1_ESM.docx]

**Supplementary figure**


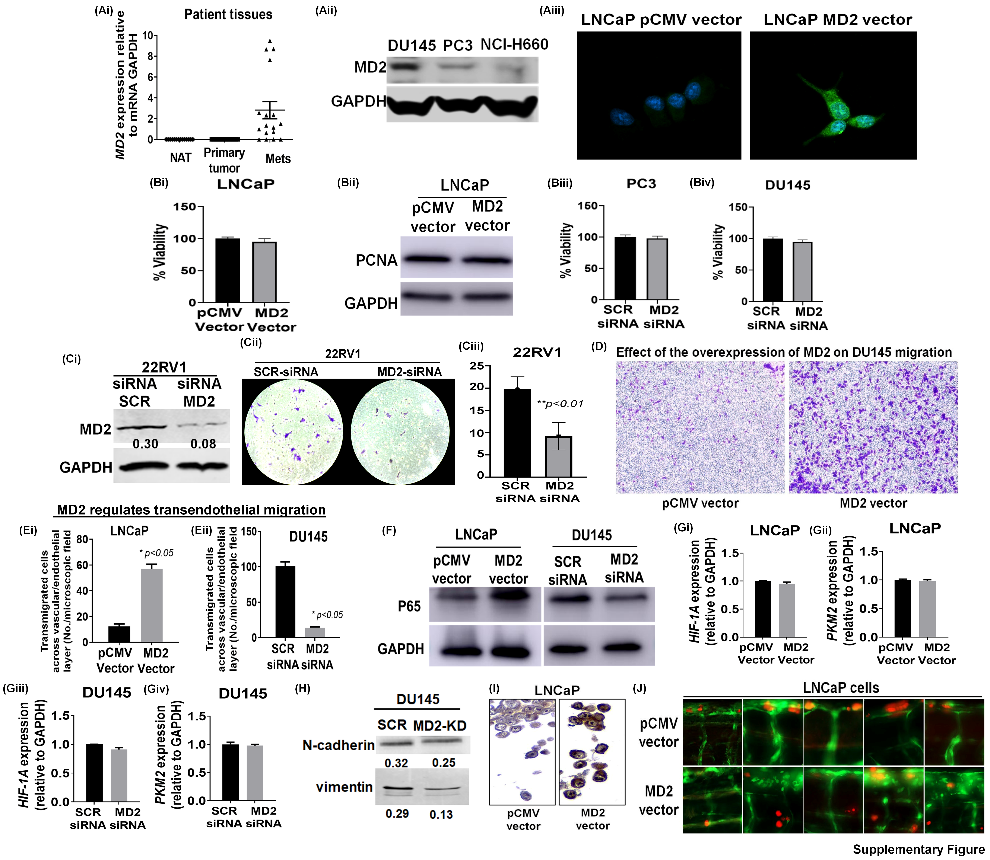


**(Ai)** *MD2/LY96* mRNA expression in patient tissues assessed by RT-qPCR. The data were normalized to GAPDH. The expression data is presented as a fold-unit change. **(Aii)** immunoblot image compares the expression of MD2 in DU145, PC3, and NCI-H660 cell lines. **(Aiii)** Image shows the detection of MD2 protein by immunofluorescence in LNCaP overexpressing MD2 (MD2 vector) and control (pCMV vector). **(Bi)** The Bar graph shows the effect of overexpressing MD2 protein on the viability of the LNCaP cells assessed at 24h by MTT assay. **(Bii)** Immunoblot image shows the effect of MD2 overexpression (LNCaP) on PCNA expression. **(Biii-iv)** Bar graphs show the effect of silencing MD2 on the viability in PC3 and DU145 by MTT assay. **(Ci)** Immunoblot image shows the silencing of MD2 protein in 22RV1 cells using siRNA. **(Cii-iii)** Image and bar graph show how silencing MD2 in 22RV1 cells affects chemoinvasion. **(D)** Images show the migrated DU145 cells overexpressing MD2 (MD2 vector) and control (pCMV vector). **(Ei-ii)** Bar graphs show the effect of MD2 overexpression (LNCaP) or silencing (DU145) on the transendothelial migration through a layer of endothelial cells (HUVEC). **(F)** Immunoblot image shows the effect of MD2 overexpression (LNCaP) and silencing (DU145) on P65 expression. **(Gi-ii)** Bar graphs show the effect of MD2 overexpression in LNCaP cells on HIF-1A and PKM2 mRNA expression assessed by RT-qPCR. **(Giii-iv)** Bar graphs show the effect of MD2 silencing in DU145 cells on the expression of HIF-1A and PKM2 mRNA assessed by RT-qPCR. **(H)** Image shows the effect of silencing MD2 in DU145 cells on the expression of the EMT markers N-cadherin and vimentin. β-actin was used as a control (showed in Fig 4A). **(H)** Detection of MD2 in LNCaP cells overexpressing MD2 (MD2 vector) and control (pCMV) by IHC. **(I)** Image shows the effect of overexpressing MD2 on the LNCaP cells' extravasation in a Zebrafish in vivo model for metastasis.
