# Supplementary material for: Myeloid differentiation factor-2/LY96, a potential predictive biomarker of metastasis and poor outcomes in prostate cancer: clinical implications as a potential therapeutic target
Source: Oncogene. 2023 Dec 23;43(7):484–94. doi: 10.1038/s41388-023-02925-x (PMC10857939; doi:10.1038/s41388-023-02925-x)
Supplement: Supplementary file 2 — Supplementary methods [file 41388_2023_2925_MOESM2_ESM.docx]

**SUPPLEMENTARY METHODS**

**Materials.** Anti-MD2, anti-VEGF, anti-human mitochondria, and anti-MMP9 antibodies were obtained from Abcam (Cambridge, MA). Anti-phospho-NFκB-p65, NFκB-p65, anti-phospho-ERK, ERK, anti-HIF-1A, anti-PKM2, anti-c-MYC, and GAPDH antibodies were purchased from Cell Signaling (Danvers, MA).

Cell culture. RWPE1 (normal), NB26 (primary tumor), 22Rѵ1 (primary tumor CRPC), and metastatic (LNCaP, PC-3 cells, and DU145) PCa cell models were purchased from ATCC and grown under standard cell culture conditions at 37^o^C and 5% CO_2_ environment. Cells were tested negative for Mycoplasma. Conditional media (CM) was prepared and collected as follows: the cells were first grown to confluence in complete culture media. Next, such cells were grown under serum-free media for 48h. The serum-free media was collected at 48h and centrifuged. The supernatant condition media was stored at -20 ^o^C until its use. A Human MD2 ELISA kit was purchased from (Thermo Fisher, Waltham, MA).

**Patient tissues.** Deidentified patient tissues were used per institutional review board (IRB)-approved guidelines and protocols. The metastatic tissues and tumor RNA of the patients diagnosed with PCa (primary tumor and metastatic tumor) were obtained from Bio-Net, an IRB-approved tissue procuring University of Minnesota facility. Serum samples from PCa patients were obtained from Bio-Net UMN.

**Patient survival analysis.** The association of *LY96* expression analysis to patient survival was determined by analyzing the TCGA-PRAD clinical dataset for PCa patients using the cBioPortal web platform.

**Patient Cohort for the Decipher-genomic test**. This study profiled RNA from primary prostate tumor specimens from patients treated with radical prostatectomy (RP) at the Department of Urology, University of Minnesota. After exclusion for tissue unavailability and quality control, the study consisted of a cohort of 228 RP-treated patients. The RNA-sequencing data was generated at the GENOME-DX facility (Genome DX biosciences, San Diego, CA). A whole-transcriptome RNA sequencing (46,000 genes & non-coding RNA) was performed. The quality control and normalization were performed using Affymetrix Power Tools and Single Channel Array Normalization (SCAN) algorithm. The GC scores are based on the predefined Decipher-classifier. The data was classified into low, intermediate, and high-risk groups. The Decipher-test data was classified, and further analysis was performed at the Institute of Informatics of the University of Minnesota.

**Transfections.** *MD2* expression was suppressed in PCa cells using a specific pool of small interfering RNA (siRNA) (Integrated DNA Technology, Coralville, IA). PC3 and DU145 cells were transfected using RNAi MAX per the vendor`s instructions (Thermo Fisher, Waltham, MA). For gene overexpression, Lipofectamine 3000 was used following the vendor’s instructions (Thermo Fisher, Waltham, MA). Lentiviral vector particles containing shRNA-MD2 were used to transfect DU145 and 22Rv1 cells according to the vendor’s instructions Santa Cruz Biotechnology (Santa Cruz, CA). Cells were selected using puromycin 2ug/ml and the silencing was confirmed by immunoblotting. The MD-2-Flag was expressed using the vector plasmid pFlag-CMV1-hMD2 purchased in Addgene a gifted by Doug Golenbock.

**Zebrafish in vivo model for metastasis.** Extravasation of tumor cells through blood vessels is required for metastasis to distant organs. We used a transgenic Tg(Fli-GFP) zebrafish, which has translucent vasculature and is ideal for tracking tumor cell movement across blood vessels. LNCaP control and LNCaP-overexpressing MD2 cells were trypsinized, counted, and labeled with Cell Tracker Orange CMTMR (Invitrogen) according to the manufacturer’s instructions. The cells were resuspended in PBS containing DNase I and heparin, and 50-200 cells were microinjected into the pericardium of anesthetized 3 dpf Tg(Fli-GFP) zebrafish, which were depigmented in embryo water containing 0.003% 1-phenyl-2-thiourea (PTU, Sigma). Embryos showing cancer cells in the bloodstream after injection were put in 34°C embryo water for 24 h and then imaged on a Zeiss Axio Observer ApoTome fluorescent microscope using standard FITC and dsRed filter sets. All zebrafish studies were conducted following institutionally approved IACUC protocols.

**Lung metastasis studies in mice.** These studies evaluated micrometastasis in the lung after tail-vein injection of tumor cells in mice. The IACUC protocol at the University of Minnesota and Rush University approved animal care and experimental protocol. According to the National Institute of Health (USA) guidelines, all animals received humane care. Male NOD.Cg-Prkdcscid Il2rgtm1Wjl/SzJ (n=40) were obtained by Jackson Laboratory. Briefly, the DU145 cells (2 x 10^6^) were injected through the tail vein using a 30-gauge needle under aseptic conditions for two consecutive days in mice weighing 25-30g. After 2 days of the second injection, mice were non-blinding and randomly distributed into SCR (n=10), MD2-DK (n=10), experimental group control: vehicle (corn oil i.p) (n=10) and group MD2-inhibitor (n=10) (5mg/kg i.p). The MD2-inhibitor treatment was administered every other day, and after the treatment protocol, mice were sacrificed on the 30th day for analysis. SCR and MD2-KD groups were maintained and euthanized for analysis on the 30th day. All animals were included in the analysis.

**Statistical analyses.** The student’s t-test for independent analysis was applied to evaluate differences between the treated and untreated cells. Statistical analyses were carried out by using PRISM statistical software. A p-value of **p< 0.05* and ***p<0.001* were considered statistically significant. To evaluate the significance of the ELISA tests and *in vivo* experiments, Mann-Whitney and Kolmogorov-Smirnov tests were applied when the data set did not show a normal distribution (ELISA test Primary tumors and MD2 metastatic group). In vivo studies: Based on our experience, the tumor intake for DU145 cells is recorded 100% in male NOD.Cg-Prkdcscid Il2rgtm1Wjl/SzJ mice. Therefore, for the statistical and power analysis consideration, we used 10 mice/ group that provides a power of >80% at 0.05 significance.
