# Supplementary material for: Myeloid differentiation factor-2/LY96, a potential predictive biomarker of metastasis and poor outcomes in prostate cancer: clinical implications as a potential therapeutic target
Source: Oncogene. 2023 Dec 23;43(7):484–94. doi: 10.1038/s41388-023-02925-x (PMC10857939; doi:10.1038/s41388-023-02925-x)

**Lymph node metastasis.** Strong to very strong immunoreactivity is seen in the nucleus of most metastatic prostate cancer cells on the left (black arrows). Stromal cells on the far left (blue arrows) are negative.


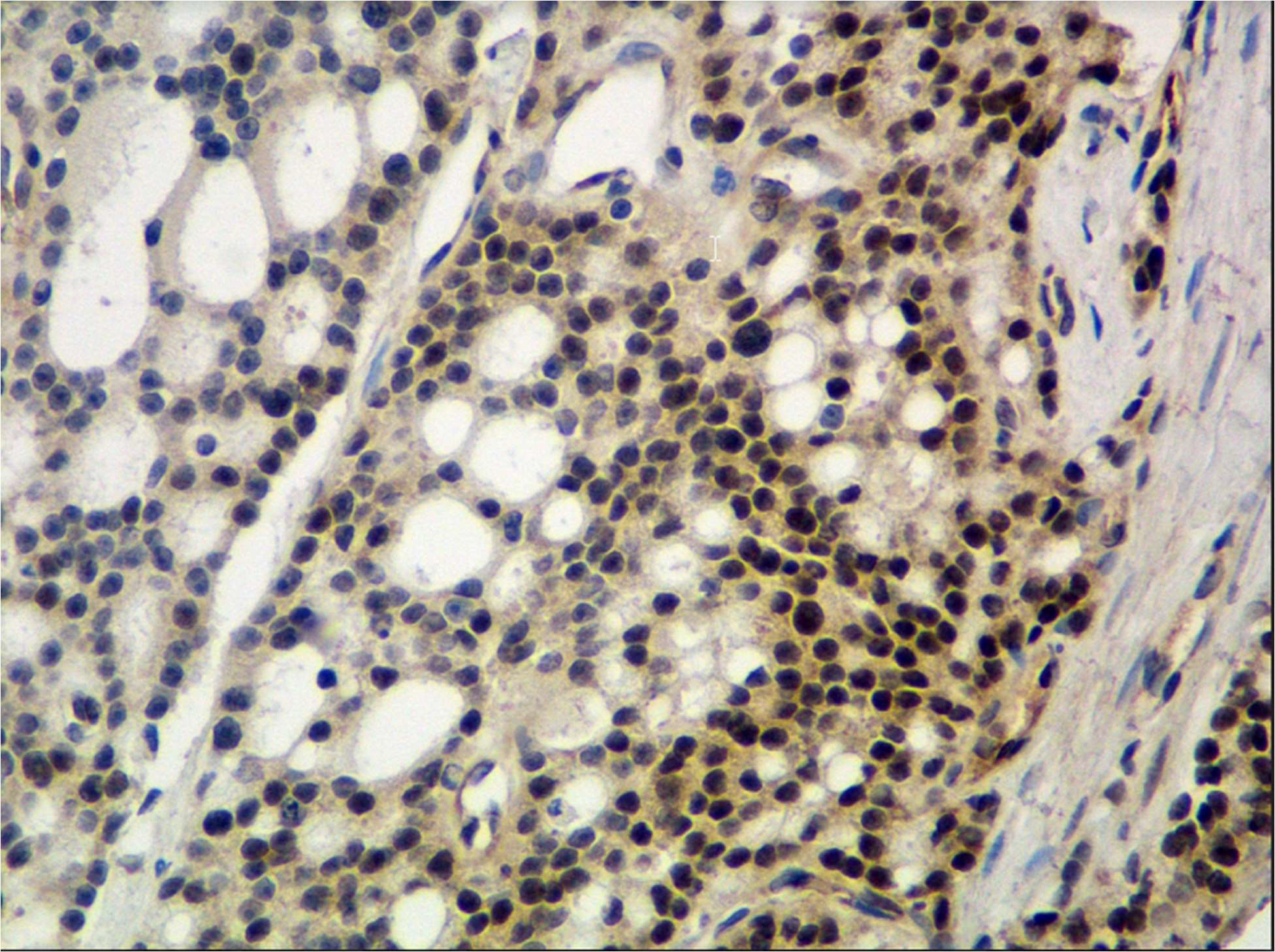


**Primary tumor (high grade)**. Moderate to strong immunoreactivity is seen in the nucleus of 80- 90% of GG3 prostate cancer cells throughout the image (example, black circle). Stromal cells (blue arrows) and vascular cells (red arrows) are negative.


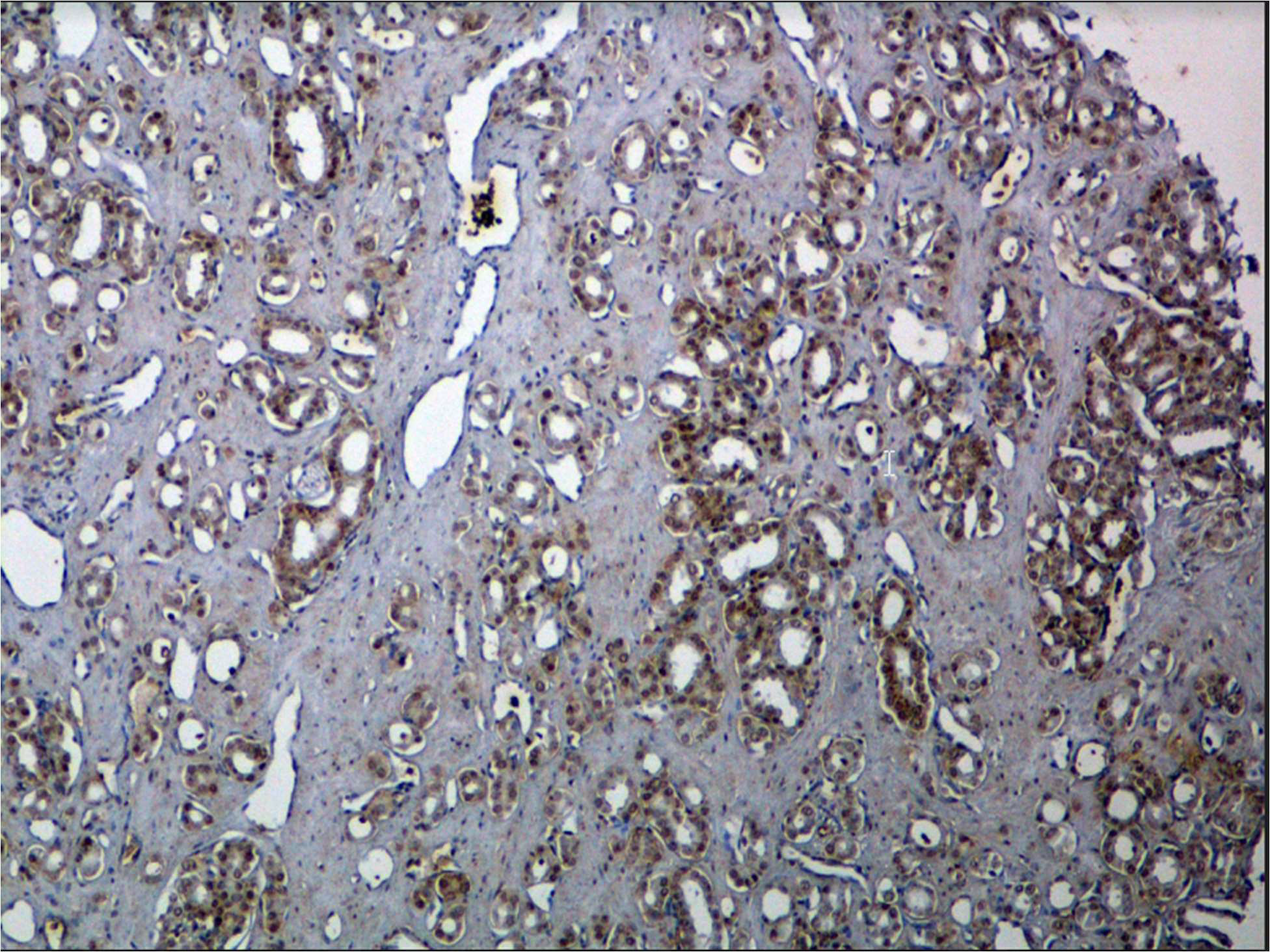


**Negative control:** Immunoreactivity is completely absent in the benign prostate glandular cells (black arrows) 20X.


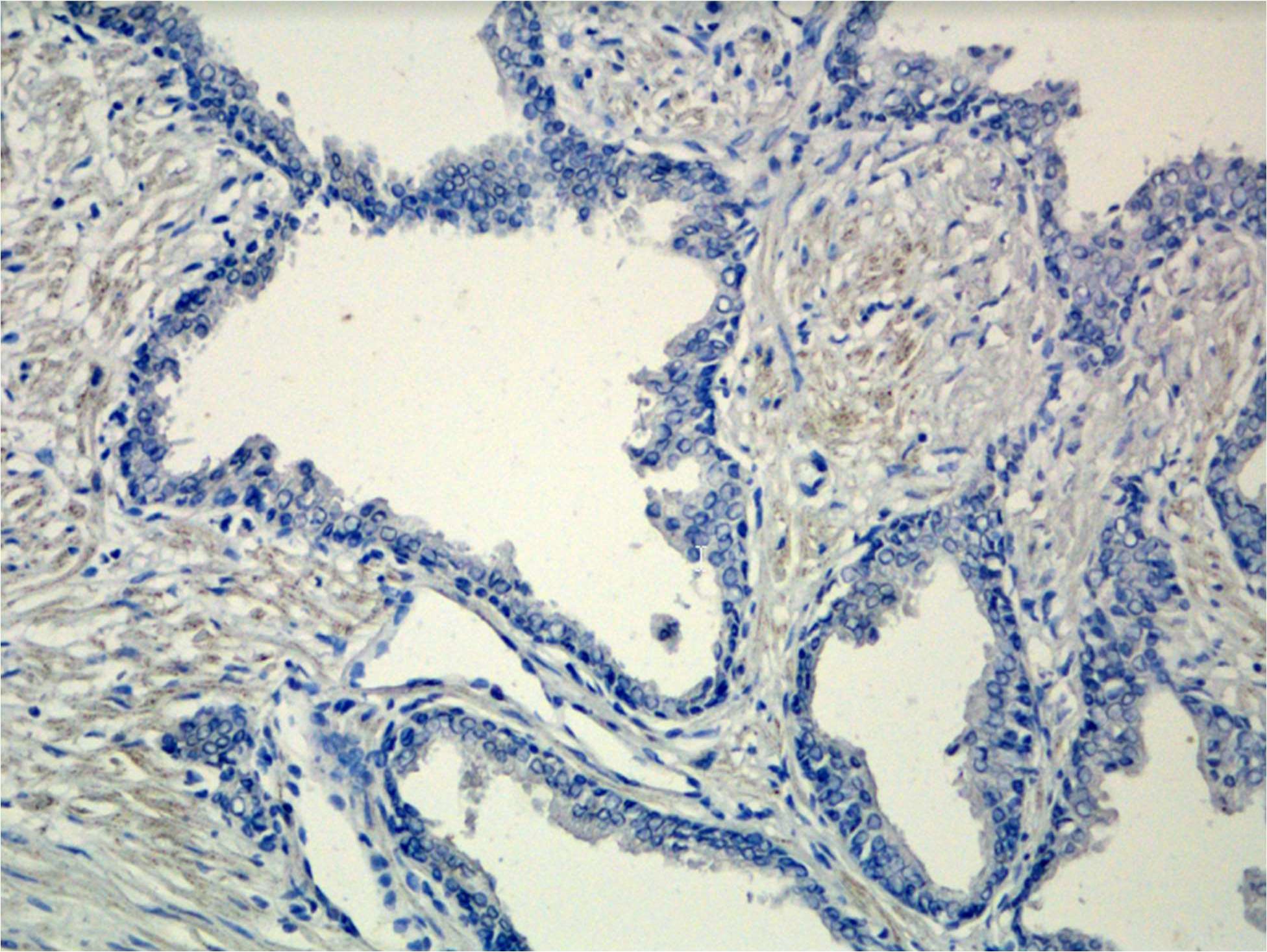


**Positive control:** Strong to very strong immunoreactivity is seen in the nucleus of all the LNCaP cells overexpressing MD2 (black arrows) 20x.


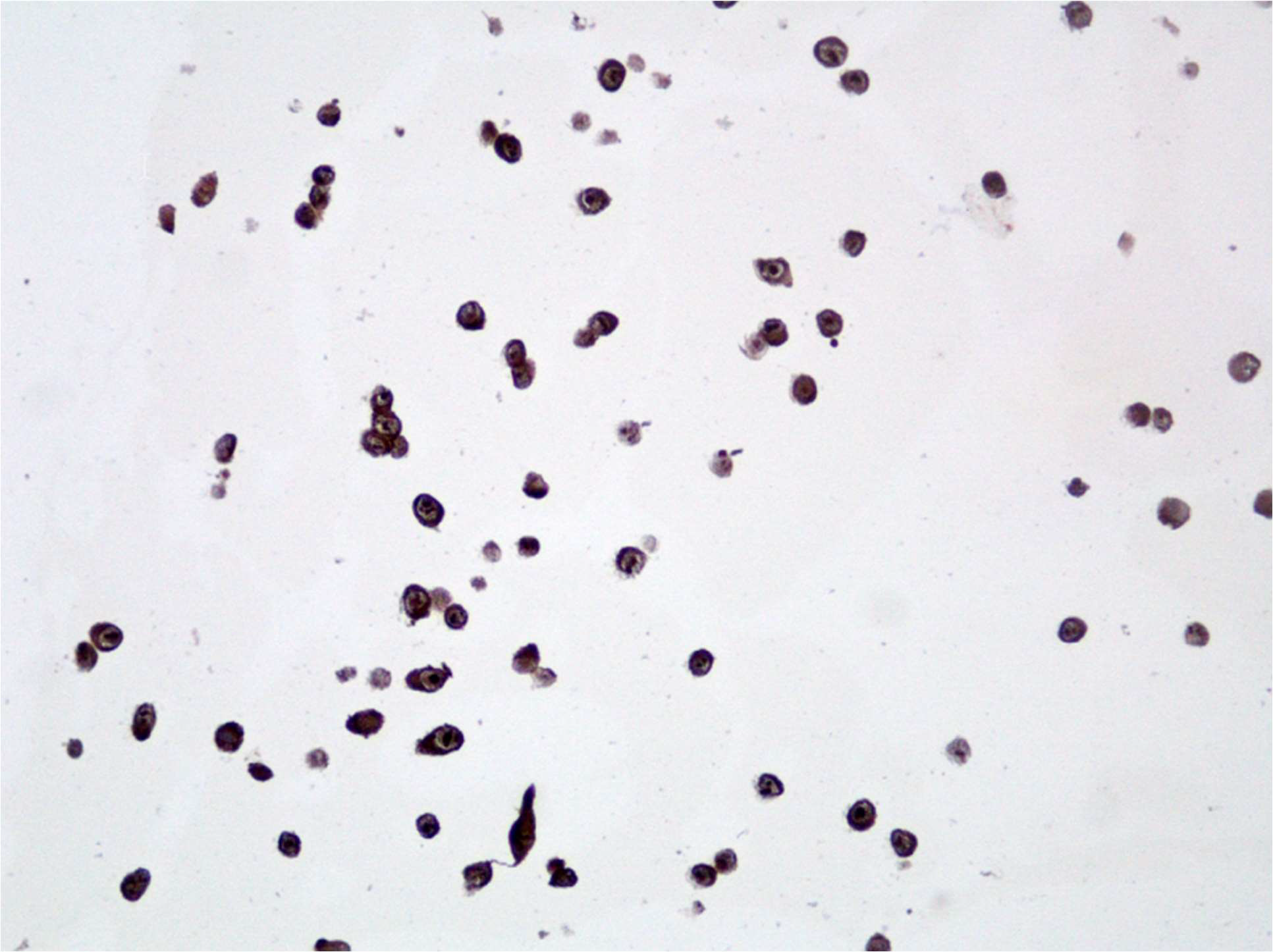

Supplement: Supplementary file 3 — Supplemantary data [file 41388_2023_2925_MOESM3_ESM.docx]
